# Conservation actions and ecological context: optimizing coral reef local management in the Dominican Republic

**DOI:** 10.7717/peerj.10925

**Published:** 2021-03-09

**Authors:** Camilo Cortés-Useche, Edwin A. Hernández-Delgado, Johanna Calle-Triviño, Rita Sellares Blasco, Victor Galván, Jesús E. Arias-González

**Affiliations:** 1 Departamento de Recursos del Mar, Centro de Investigación y de Estudios Avanzados del I.P.N, Mérida, Yucatán, México; 2 Wave Of Change, Iberostar Hotels & Resorts, Playa Paraíso, Quintana Roo, México; 3 Deparment of Environmental Sciences, University of Puerto Rico, San Juan, Puerto Rico; 4 Center for Applied Tropical Ecology and Conservation, University of Puerto Rico, San Juan, Puerto Rico; 5 Sociedad Ambiente Marino, San Juan, Puerto Rico; 6 Fundación Dominicana de Estudios Marinos, Bayahibe, La Altagracia, Dominican Republic

**Keywords:** Coral reefs, Coral restoration, Management, Coastal health, Marine protected area, Water quality, Tropical coastal ecosystems, Biodiversity, Dominican Republic, Caribbean

## Abstract

Over the past few decades, coral reef ecosystems have been lost at accelerated rates as a result of global climate change and local stressors. Local management schemes can help improve the condition of coral reefs by enhancing their ecosystem recovery capacity. Caribbean conservation efforts include mitigation of local anthropogenic stressors, and integrating social participation. Here, we analyzed the case of the Bayahibe reefs in the Southeastern (SE) Dominican Republic to identify conservation actions and illustrate a conceptual example of local seascape management. We assessed reef health indicators from 2011 to 2016. Overall, our results show increases in total fish biomass, in both commercial and herbivorous fishes. Mean live coral cover was 31% and fleshy macroalgae was 23% after multiple disturbances such as Hurricanes Sandy and Isaac (2012), Mathew (2016) and heat stress presented in the study area in 2015. We also described actions taken by stakeholders and government institutions, including the implementation of a policy declaring an area of 869,000 ha as a marine protected area (MPA), enhanced water quality treatment, local restrictions to vessel traffic, enforcement of fishing regulations, and the removal of invasive lionfish (*Pterois* spp.). In addition, a restoration program for the threatened staghorn coral (*Acropora cervicornis*) was established in 2011, and currently has eight coral nurseries and six outplanting sites. Considering the biology and ecology of these reefs, we observed good results for these indicators (live coral cover, fish biomass, and water quality) in contrast with severely degraded Caribbean reefs, suggesting that optimizing local management may be a useful example for improving reef condition. Our results provide an overview of trends in reef condition in the SE Dominican Republic and could support current strategies to better protect reefs in the region. Given that Caribbean coral reefs face extreme challenges from global climate change, management measures may improve reef conditions across the region but stronger policy processes and increased scientific knowledge are needed for the successful management of coral reefs.

## Introduction

Coral reefs are important ecosystems commonly found in tropical and subtropical Small Island Developing States (SIDS) (Wilkinson et al., 2016). Millions of people around the world depend on the numerous ecosystem services that coral reefs provide (Cinner et al., 2016). In the Dominican Republic, for example, coral reefs serve as a source of protein for an estimated 14,500 artisanal fishermen which contribute an estimated US$51.6 million to the country’s GDP (Herrera-Moreno et al., 2014). Despite the numerous services that coral reefs provide, recent evidence from urban and rural coastal zones suggests coral reefs are suffering the impact of direct human activities such as pollution, overfishing, and habitat deterioration (Hughes et al., 2003; Anthony et al., 2015). Poor implementation of sustainable development across tropical islands can also result in sustained impacts on coastal water quality, affecting coral reefs and other coastal ecosystems (Ramos-Scharrón, Torres-Pulliza & Hernández-Delgado, 2015; Hernandez-Delgado et al., 2017). Recently in the Caribbean region, wastewater and groundwater discharges have been associated with phase shifts from coral-dominated reefs to algal-dominated reefs (Arias-González et al., 2017; Otaño-Cruz et al., 2017) and with chronic declines in reef-building species assemblages (Díaz-Ortega & Hernández-Delgado, 2014; Ware et al., 2020). In addition, Sims et al. (2020) mentioned that nutrient enrichment by groundwater discharge could be an important cause of negative change for coral communities at near-shore reef sites.

Coral reefs are also suffering from the impacts of natural phenomenons (e.g., hurricanes, storms) and/or global climate change (e.g., changes in temperature, pH and O_2_) (Hoegh-Guldberg et al., 2007; Hughes et al., 2017a). These multiple disturbances combined with the lack of local regulations and poor governance (Bozec & Mumby, 2015), may result in a loss of coral cover (Gardner et al., 2003) and biodiversity (Pandolfi et al., 2003), as well as changes to food webs and habitat structure (Micheli et al., 2014). Thus, coastal populations are losing important goods and services (Moberg & Folke, 1999), including food and medicinal products, protection from the damage caused by natural phenomena (Spalding, Corinna & Green, 2001), and income provided by tourism (Wilkinson & Souter, 2008). Considering the importance of coral reefs and global change impacts, their current status requires data-driven goals to inform decision-makers and novel reef management actions to support their conservation (Halpern et al., 2012; Allan et al., 2013; Camacho, Steele & Challenger, 2020). Several conservation interventions have been proposed in this context, including the establishment of marine protected areas (MPAs), No-take zones, constrained fishing, pollution reduction measures, and coral restoration activities (Duarte et al., 2020). These local actions may protect habitats, increase coral cover, aid in the recovery of fisheries productivity, and move towards operationalizing resilience to disturbances (Nyström et al., 2008; Mumby et al., 2014).

**Figure 1:**
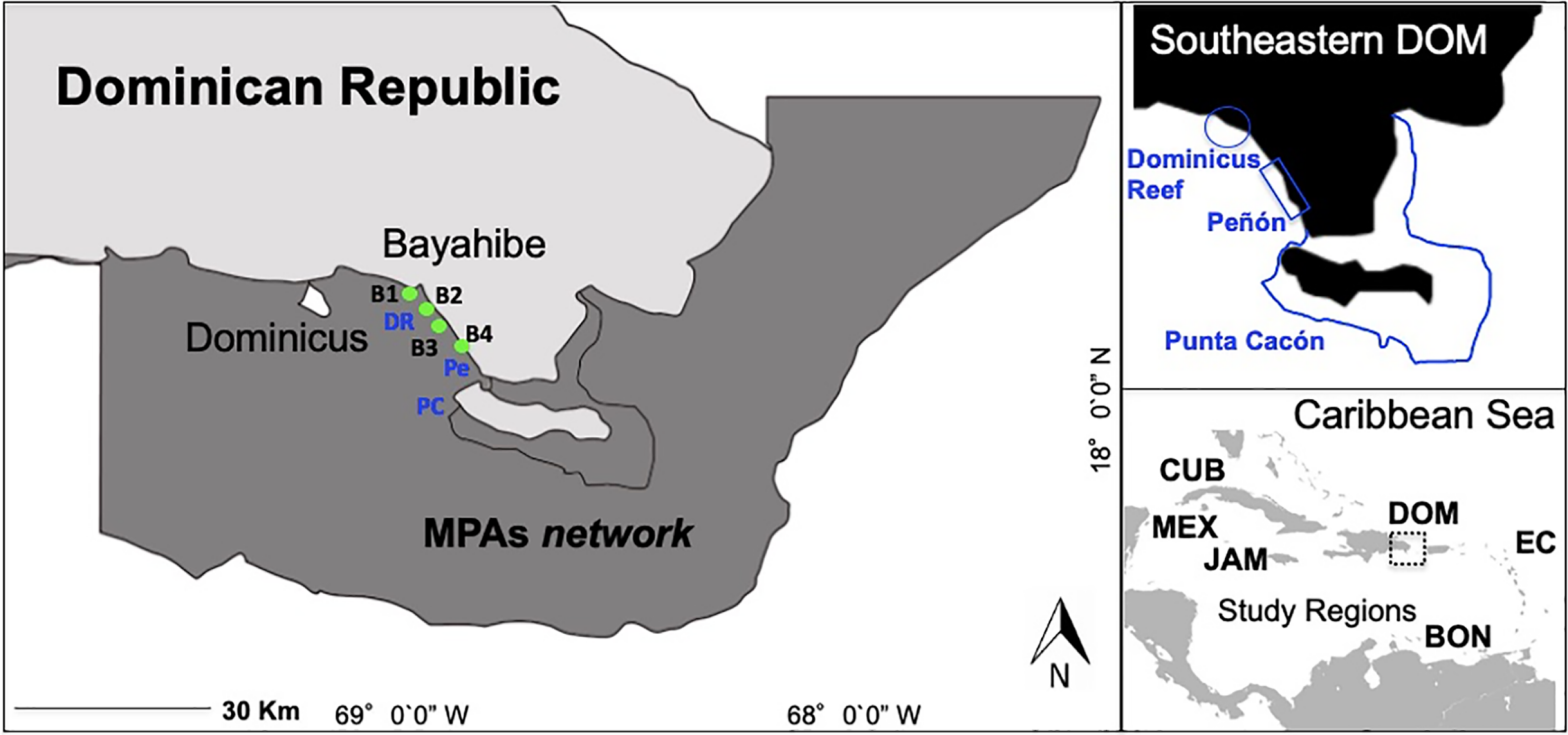
Southeastern Dominican Republic, Caribbean. Blue lines display the ecological sampling sites located in MPAs network: DR = Dominicus Reef (Southeastern Coral Reef Marine Sanctuary), Pe = “Peñón” reef (Guaraguao Catuano Recreation Natural Area) and PC = “Punta Cacón” reef (Cotubanamá Natural Park). Green dots display water quality sampling sites B1, B2, B3 and B4 located in tourism areas (Bayahibe-Dominicus). Inset rectangle shows study regions used for comparison, including Mex, Mexico; Cub, Cuba; Jam, Jamaica; Dom, Dominican Republic; Bon, Bonaire; EC, Eastern Caribbean islands.

Here, we focus on the community of Bayahibe in the Southeastern Dominican Republic on the island of Hispaniola as a case study of the impacts of local management interventions on coastal ecosystems (Fig. 1). Our ecological context framework is based on an extensive Caribbean literature review and uses a local management approach (Chamberland et al., 2017). The basis of our case study includes an implementation of active actions combined with passive actions for areas of high tourism on Bayahibe. We report on actions funded and carried out by several non-governmental organizations (NGOs), diving centers, private sector institutions, local communities, and government authorities. Actions implemented included No-take zones enforced since 2009, water treatment plants by hotels based on environmental criteria for Blue Flag certified beaches, removal of invasive or predatory species, and a coral restoration program that includes asexual and sexual propagation, and enhancing genetic species diversity (Cortés-Useche et al., 2017; Calle-Triviño et al., 2017, 2018). Stakeholders in Bayahibe have been actively involved since 2011 in removing invasive species (*Pterois* spp.), installing mooring buoys and navigational signage at reefs sites, and permanently controlling, surveilling and monitoring the area.

We documented the condition of selected coral reefs (using multiple reef health indicators) in the Bayahibe area during the period from 2011 to 2016 and report on a multi-spectrum of analyses for coastal management processes and their implementation benefits on coral reef condition. To our knowledge, this is the first contextual analysis on local marine management in the Bayahibe area. We illustrated this framework by presenting conservation actions at the local level that can be adapted to coral reefs management in the specific situation of intense coastal development and tourism activities. We compared our study considering reef health indicators and management status with other sites across the Caribbean region with similar physical characteristics and survey methods (Jackson et al., 2014; Perera-Valderrama et al., 2016; Steneck et al., 2018, 2019; Cortés-Useche et al., 2019; Martínez-Rendis, Acosta-González & Arias-González, 2020) (Table 1). The aim of our study was to describe a conservation case using an ecological context framework and make information available on the restorative actions, active and passive, being implemented at the local level for the improvement of coral reef health.

**Table 1:** List of codes, status and reef health indicators of the 28 study sites in Caribbean region used for comparison.

Study region	Site	Code	Status	Reef health indicators				References
				CC	FMC	PB	CB	
Dominican Republic	Dominicus reef	DR	PP	✓	✓	✓	✓	This Study, 2021
	Peñón reef	Pe	PP	✓	✓	✓	✓	
	Punta Cacón reef	PC	PP	✓	✓	✓	✓	
	Boca Chica	BC	UP	✓	✓	✓	✓	Cortés-Useche et al. (2019)
Cuba	Yemaya	Ye	PP	✓	✓	X	X	Perera-Valderrama et al. (2016)
	Laberinto	La	PP	✓	✓	X	X	
	Jardines de la Reina	JDR-NTZ	NTZ	✓	✓	✓	X	Jackson et al. (2014)
Jamaica	Montego Bay	MB	PP	✓	✓	✓	X	
	Port Royal Cays	PRC	PP	✓	✓	X	X	
	West	We	PP	✓	✓	✓	X	
Mexico	Cozumel	C-NTZ	NTZ	✓	✓	✓	✓	Martínez-Rendis, Acosta-González & Arias-González (2020)
Bonaire	Bonaire	B-NTZ	NTZ	✓	✓	✓	X	Steneck et al. (2019)
Eastern Caribbean	Anguilla	An	UP	✓	✓	✓	✓	Steneck et al. (2018)
	St. Marteen	SM-NTZ	NTZ	✓	✓	✓	✓	
	St. Croix	SC	UP	✓	✓	✓	✓	
	St. Croix	SC-NTZ	NTZ	✓	✓	✓	✓	
	Barbuda	Ba	UP	✓	✓	✓	✓	
	Antigua	At	UP	✓	✓	✓	✓	
	St.Lucia	SL	UP	✓	✓	✓	✓	
	St.Lucia	SL-NTZ	NTZ	✓	✓	✓	✓	
	Bequia	Be	UP	✓	✓	✓	✓	
	Mustique	M-NTZ	NTZ	✓	✓	✓	✓	
	Canoan	Ca	UP	✓	✓	✓	✓	
	Tobago Cays	TC-NTZ	NTZ	✓	✓	✓	✓	
	Union & Pt St. Vin	Un	UP	✓	✓	✓	✓	
	Carriacou	Cr	UP	✓	✓	✓	✓	
	Grand Ave	GA	UP	✓	✓	✓	✓	
	Grand Ave	GA-NTZ	NTZ	✓	✓	✓	✓	

**Note:**

PP, partially protected areas; UP, unprotected; NTZ, No-take zone; CC, Coral Cover; FMC, Fleshy Macroalgae Cover; PB, Parrotfish (Scaridae) Biomass; CFM, commercial fishes (Lutjanidae and Serranidae) Biomass.

## Materials and Methods

### Study area

The study was conducted at Bayahibe, a municipality on the Dominican Republic’s South-eastern Caribbean coast (Fig. 1) with a huge influx of tourism. Coral reef sites are distributed along a semi-continuous fringing reef dominated by rocky and coral substrate, with small and dispersed coral patches (Geraldes, 2003). The municipality is part of the Romana–Bayahibe–Dominicus tourism destination area, which is characterized by a fast transformation of the coastline and coastal seascape during the last decades (Cortés-Useche et al., 2019). It is currently a leading tourist attraction for the Dominican Republic, with an average of approximately 559,000 visitors per year and over 3,300 hotel rooms (Herrera-Moreno et al., 2014).

The Bayahibe area includes several MPAs traditionally managed from a cultural/tourism perspective (Herrera-Moreno et al., 2014), with multiple landscape and seascape protection that include (1) Cotubanamá National Park (CNP) which was established in 1975 by a top down government mandate (796.40 km^2^), and included within, a No-take reserve in the reef lagoon between the mangrove forests and Saona Island, close to “Punta Cacón” reef, called a “Catuano” channel where fishing and vessel traffic has been prohibited since 2009 by Decree 499‒09 (Cortés-Useche et al., 2019), (2) Guaraguao Catuano Natural Recreation Area (GCNRA) established in 1975, that occupies a marine area of 18.59 km^2^ and includes land-based protection of important ecosystems such as seagrass and coastal dunes (SINAP, 2014), and (3) the Southeastern Coral Reef Marine Sanctuary (SCRMS) with an area of 7,855.31 km^2^, declared in 2009 as an MPA by Dominican Government Decree 571-09. The main goal of these designations was to conserve natural habitat and the unique environment that exists along the continental shelf on the SE part of the Hispaniola Island, and includes Dominicus reef sites linked to a thriving snorkeling and diving tourism (Table S1; Cortés-Useche et al., 2019).

### Data collection and statistical analyses

#### Coral reef condition

Ecological data was gathered on reef condition during the period from 2011 to 2016 from regional datasets in coordination with the Dominican Foundation for Marine Studies (FUNDEMAR) (Cortés-Useche et al., 2017, 2018, 2019). The majority of the data consisted from underwater visual census (UVS) carried out with SCUBA at approximately 10 m depth. The point intercept transect method in permanent stations was used to collect benthos data. Visual counts along belt transects were used to collect fish data. Datasets were collected by surveyors working in teams of five. All surveyors were trained and certified in the Atlantic and Gulf Rapid Reef Assessment (AGRRA) methodology by AGRRA instructors and in SCUBA by certified trainers from the Professional Association of Dive Instructors (PADI). Each fixed transect (6) for the benthos data collection was 10 m in length with data points collected every 10 cm along the transect, giving a total of 100 points per transect. For fishes, visual counts were recorded along belt transects (30 m × 2 m each) located around the habitat used for benthos transects (Lang et al., 2010). Annual monitoring was conducted at these sites during the period of May–August. Ecological Assessment was based on ecological indicators: mean live coral cover and mean fleshy macroalgae cover, and reef fish biomass (commercial and herbivorous fishes). Benthic and fish communities were monitored in three fringing reefs in the Bayahibe area distributed in the three different MPAs by the Ministry of Environment and Natural Resources: (1) Cotubanamá Natural Park (“Punta Cacón” = PC), (2) Guaraguao Catuano Recreation Natural Area (“Peñón” = Pe), and (3) Southeastern Coral Reef Marine Sanctuary (Dominicus Reef = DR). Benthic community raw data was converted to cover and abundances percentage for each benthic category type; fish abundances and fork lengths were used to calculate total fish biomass using length-weight relationships. Spatial (sites) and temporal factor (years) variation were evaluated using a permutational multivariate analysis of variance (PERMANOVA) and pairwise comparison. An additional comparison was added between community structure in 2011 (pre-disturbances) and 2016 (post-disturbances). This comparison was motivated to assess reef health and evaluate local management processes. A principal coordinate ordination (PCO) analysis was performed, by calculating the distance among centroids to display the variations in coral species composition in a three-dimensional space and determine which species explained spatio-temporal variation. Statistical analyses were carried out with PRIMER v6 and PERMANOVA v1.16 statistical programs (Anderson, Gorley & Clarke, 2008).

#### Water quality

Four water quality-sampling stations were selected along the high tourism area of the Bayahibe-Dominicus coast (B1, B2, B3 and B4) to assess water quality parameters. The study sites were located in a gradient of tourism intensity near the coast (<2 km) and close to the DR site. Between 2011 and 2016, seasonal sampling for environmental variables was conducted (dry and wet seasons) with sea surface temperature (SST), pH, and turbidity (in nephelometric turbidity units = NUT) triplicate measures. Data was obtained using a U52G Horiba Water Meter (Horiba Instruments, Kyoto, Japan) and a Hach portable turbidity meter. Sampling was conducted taking into consideration that the seasons of the year are influenced by prevailing wind direction, air temperature and precipitation during July–November (wet), December–February (dry), and March–June (transition) (Chiappone, 2001). To link microbial water quality (WQ) data and reef health data we used as a reference site B4 and DR site (high impact of tourism). Three microbiological indicators (total coliforms (TC), fecal coliforms (FC) and Enterococci (ENT)) were measured in-situ at 10 m depths in triplicates. Samples of comparable water were collected for laboratory analyses. Grab samples were analyzed following standard membrane filtration techniques by American Public Health Association (APHA) to quantify total coliforms (TC), fecal coliforms (FC) and Enterococci (ENT) following Bonkosky et al. (2009) and Otaño-Cruz et al. (2017). Colonies concentration was expressed in colony forming units (CFU). Standard methods for the examination of water and wastewater were used (Díaz-Ortega & Hernández-Delgado, 2014). WQ components (environmental variables and microbiological indicators) were tested using non-parametric permutational analysis of variance (PERMANOVA) and pairwise comparison for the fixed factors of seasons, time (year) and site. An additional analysis was added to observe the temporal dynamic of the WQ pulse event by interaction of season by factors (WQ components) (Anderson, Gorley & Clarke, 2008). BEST BIO-ENV and RELATE (Spearman rank) correlations, were calculated to correlate the WQ and reef health spatio-temporal variation for DR site, the one with highest tourism development (Clarke et al., 2014). This was done to identify important predictors of ecological condition in response to WQ in DR site. Analyses were carried out with PRIMER v6 and PERMANOVA v1.16 statistical programs (Anderson, Gorley & Clarke, 2008).

#### Control of invasive species

As part of the management actions, removal of invasive lionfish (*Pterois* spp.) was funded and carried out by FUNDEMAR and the local community. The main goal of the removal through the implementation of tournaments (derbies) was to reduce local population growth and spread of lionfish facilitated by public participation (Malpica-Cruz, Chaves & Côté, 2016). Lionfish removal efforts in Bayahibe were focused on frequently visited sites by dive operators and artisanal fishers. All sites were <30 m depth. Since 2011, annual derbies have been performed during the December season. Teams consisted of a mixed public, including artisanal fishers, sport fishers, divers and local volunteers using freediving or scuba diving, depending on depth. Fish were captured with spearguns and Hawaiian slings. Subsequently, captured individuals were counted and measured (total length) at Bayahibe Harbor. One-way PERMANOVA was used to determine significant differences in lionfish abundance (No. of individuals) between annual removal events (Anderson, Gorley & Clarke, 2008). Catch and effort data was analyzed to calculate catch per unit effort (CPUE) (Individuals/unit/year) of lionfish caught during the annual derbies. Abundance data was square root transformed. Analyses were conducted using Bray–Curtis dissimilarities and 9,999 permutations.

#### Coral reef restoration

In Bayahibe there is an established and matured *Acropora cervicornis* coral restoration program currently composed of 1 primary and 7 secondary nurseries as well as six transplant sites. We analyzed data presented by Calle-Triviño et al. (2020) of a temporal assessment of the coral restoration program since its implementation in 2011–2017 to address the active management process. In that assessment, Calle-Triviño et al. (2020) used the methodology proposed by Schopmeyer et al. (2017) to determine restoration success by evaluating the growth (expressed as Total Linear Extension in cm), survival (determined by counting the number of colonies with some percentage of living tissue at the start of the study, and then 12 months later), and productivity (determined by the following formula: Annual productivity = (growth at T(final)/initial TLE)) of colonies installed in the nurseries and outplanted sites. From this, Calle-Triviño et al. (2020) proposed reference points for measuring the first year of restoration: (1) the survival of corals in the nursery must be greater than 80%, and (2) the survival of outplanted corals must be greater than 70%. Average productivity should be >4.4 cm year^−1^ for corals in nurseries and >4.8 cm year^−1^ for outplanted corals. Genetic diversity was also determined for *A. cervicornis* in the main nursery. Genotyping was performed using a DNA IR24300 sequencer (Li Cor, Lincoln, NE, USA) and diversity genetic index was estimated using GenClone software v. 2 and GenAlEx v 6.4 to discriminate distinct multilocus genotypes (MLGs). Calle-Triviño et al. (2020) also presented a preliminary analysis of the strong cyclonic seasons that struck the Greater Caribbean region in 2016 and 2017 which we analyze here in more detail.

## Results

### Reef condition

**Figure 2:**
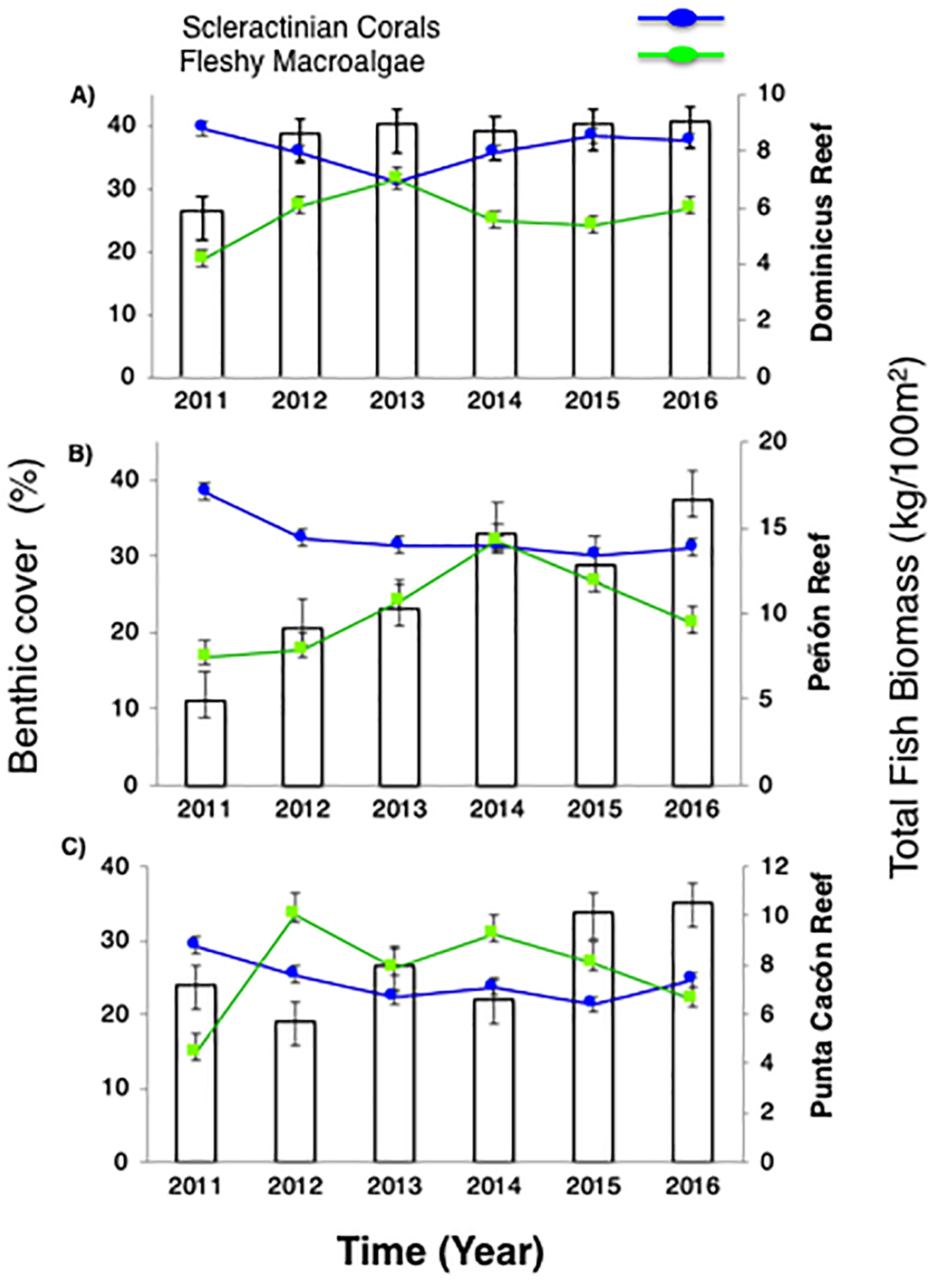
Trends in the reef health indicators. (A) Dominicus Reef (Southeastern Coral Reef Marine Sanctuary), (B) “Peñón” reef (Guaraguao Catuano Recreation Natural Area) and (C) “Punta Cacón” reef (Cotubanamá Natural Park). White bars represent total fish biomass (TB) (mean and standard errors). Blue line and dots show live coral cover. Green line and dots represent fleshy macroalgae cover (mean and standard errors).

Live coral percent (%) cover for selected reefs in Bayahibe ranged from 21% to 39% with an average of 31% (±1.04 SE) during the period from 2011 to 2016 (Table S2). The spatio-temporal analysis showed no significant differences (*P*-value = 0.875, Table S3), among the three sites in 2011 and 2016, following multiple disturbances such as Hurricanes Sandy and Isaac (2012), Mathew (2016) and heat stress presented in the study area in 2015. Mean coral cover values changed relatively little over the study period from 39% to 37% in DR, 38% to 31% in Pe, and 29% to 25% in PC (Fig. 2). This is consistent with the fact that no significant differences in coral coverage were observed among years (*P*-value = 0.139, Table S3). The main contributor to live coral cover in the Bayahibe reefs was *Orbicella* spp. complex and *Agaricia agaricites*. These species represented 31% and 24% of documented live coral cover respectively (Table S4; Cortés-Useche et al., 2019). Analysis of the post-disturbance data hurricane events 2012, 2016 and thermal stress 2015, showed that the benthos continued to be dominated by reef-building species. At the DR site, we found a seascape dominated by *Montastraea cavernosa*, the Pe site was dominated by *Orbicella faveolata*, while in the PC site *Pseudodiploria strigosa* dominated the seascape (*P*-value = 0.001, Table S3). Indeed, according to the PCO analysis accounted for 55.2% of total spatio-temporal variance (Fig. S1).

Further, fleshy macroalgae percent (%) cover at the same sites ranged from 14% to 36% with an average of 23% (±1.78 SE). There was a noticeable increase in % cover in 2012 across the three sites but remained stable afterwards, until 2015. FMC showed no significant temporal patterns changes among years (*P*-value = 0.869, Table S3). Nevertheless, increases were noticeable between2011 and 2016 (*P*-value = 0.0334, Table S3). By 2016, macroalgal assemblages were dominated by *Dictyota* spp. and *Halimeda* spp. FMC values at DR were 27% followed by PC 22% and Pe 21% (Fig. 2). No spatial patterns differences (between the three sites) in algal assemblage structure were observed (*P*-value = 0.144, Table S3).

Despite total fish biomass (TB) showing no significant temporal patterns (*P*-value = 0.076, Table S3), total fish biomass and population abundances improved noticeably from 2011 to 2016 (*P*-value = 0.0001, Table S3). By 2012, both biomass and abundance increased progressively until 2016 when fish biomass reached twice that recorded in 2011. The observed increase for DR was from 5.8 to 9.1 kg/100 m^2^, at Pe from 4.9 to 16.6 kg/100 m^2^, and at PC from 7.2 to 10.5 kg/100 m^2^ (Fig. 2).

**Figure 3:**
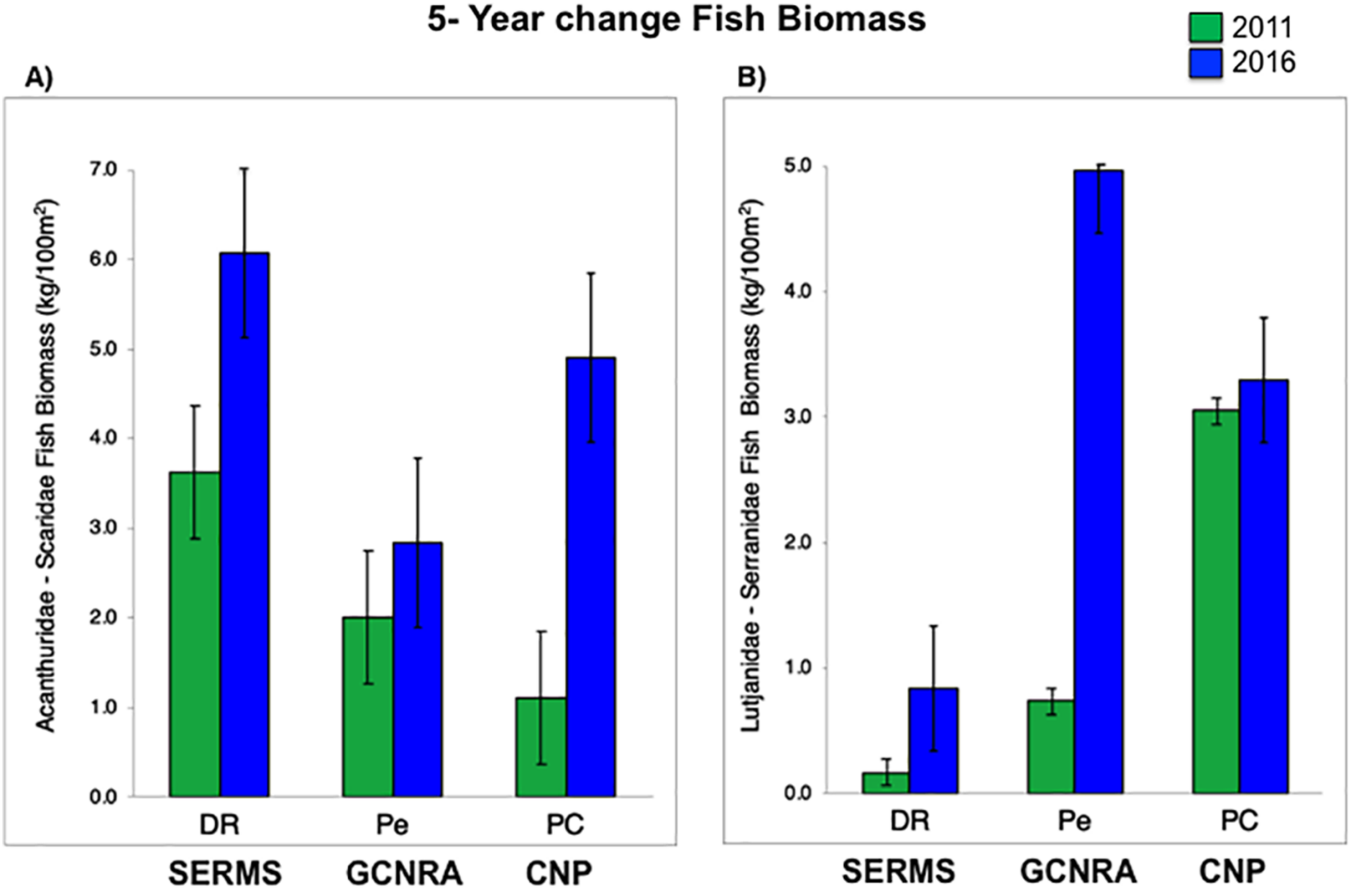
(A) Fish biomass (mean and standard errors) recovery for herbivorous (Acanthuridae and Scaridae) and (B) commercial fishes (Lutjanidae and Serranidae) period from 2011 to 2016, (mean and standard errors). CNP, Cotubanamá Natural Park; GCNRA, Guaraguao Catuano Natural Recreation Area; SCRMS, Southeastern Coral Reef Marine Sanctuary.

For herbivorous fishes (Acanthuridae and Scaridae), biomass increased in the three study sites from 2011 to 2016 (*P*-value = 0.0007, Table S3). DR increased from 3.6 to 6.1 kg/100 m^2^, Pe from 2 to 2.8 kg/100 m^2^, and PC from 1.1 to 4.9 kg/100 m^2^. Commercial fish (Lutjanidae and Serranidae) increased in the same way (*P*-value = 0.0153, Table S3), at DR from 0.2 to 0.8 kg/100 m^2^, at Pe from 0.7 to 5 kg/100 m^2^, and at PC from 3 to 3.3 kg/100 m^2^ (Fig. 3). This increasing spatial trend is consistent with the statistically obtained by PERMANOVA between the three sites (*P*-value = 0.157, Table S3).

### Water quality

Overall, no significant spatio-temporal (seasons, time, and sites) differences were observed for WQ components (*P*-value > 0.05, Table S3). However, the interaction of season by factors (WQ components) analyzed in this study showed significant differences (*P*-value < 0.05, Table S3), except for ENT concentration (*P*-value = 0.1499, Table S3), that presented values of <10 cfu/100 mL for all sites and a mean of 2 cfu/100 mL. For TC, values (*P*-value = 0.1289, Table S3), t ranged from 14 to 50 colony forming units (cfu/100 mL) at the different stations. For FC, (*P*-value = 0.0271, Table S3), mean concentration was 12 cfu/100 mL for all observations. In addition, during the period from 2011 to 2016, SST values ranged from 26.9 °C to 30.5 °C. For example, seasonal trends showed SSTs were highest during the late summer months (September and October; ~30.5 °C), and the lowest were recorded from January to March (~26.9 °C) (*P*-value = 0.0001, Table S3). pH values ranged from 7.9 to 8.7 (*P*-value = 0.0001, Table S3), while mean turbidity values were low in sampled sites (<3 NTU). However, the results showed significant variation in NTU with higher values in the wet season of 2012 and 2015 and dry season of 2014 (>5 NTU) (*P*-value = 0.0001, Table S3). B4 site showed the high values for the zone with extreme values in wet season 2014 and dry season 2015 (>13 NTU). The non-parametric correlation BEST BIOENV (Spearman rank) analyses identified a group of WQ variables that had a weak correlation with coral reef health components, composed of TCO, ENT and NUT (Rho = −0.171) by time series.

### Control of invasive species

A total of 2,534 lionfish (*Pterois* spp.) individuals were captured during the 2011–2016 period. Sizes ranged from 4.2 to 42.3 cm (LT) with an average of 20.5 cm. The average size class in the first year (2011) was 18 cm and 25 cm in the sixth year (2016). On average, 422 lionfish individuals were captured per year. The greatest number of captured fishes occurred in 2014 (469) and the lowest number (384) in 2012. The best catch per unit effort occurred in 2014 with 13.5 (ind/unit). Our results suggest greater lionfish abundances in the 2014 as catch was greatest even with lower participant involvement compared to other years. Results demonstrate constant capture (*P*-value = 0.834); no significant temporal differences were observed for the number of lionfish individuals during the period from 2011 to 2016. However, modal progression to capture larger individuals was observed, with the highest peaks in 2015 and 2016 (Fig. S2).

### Coral reef restoration

The coral restoration program in Bayahibe by 2017 had a total of eight Acropora nurseries established (1 main nursery and 6 secondary nurseries) with a cumulative live tissue total of 26,000 linear cm (TLE). Equivalent to more than 1,400 fragments distributed across a wide range of sizes (<25 cm = 163, 26–50 cm = 162, 50–100 cm = 179, 100–200 cm = 250 and >200 cm = 66), with high survival (>80%), and annual productivity values >4.4 cm per year. Additionally, the program had six outplanting sites with 1,446 transplanted colonies, high survival rates (>70%) and annual productivity of 4.8 cm year^−1^. The mean survival of all nursery fragments after the 2016 and 2017 cyclonic seasons was 35.06 ± 11.30%, with a range of 16.96–52.07%. The mean survival of the outplanted colonies in four outplanted sites operating after Hurricane Matthew (2016) was 28.68 ± 20.0%, with a range of 5.49–51.78%. The mean survival of the outplanted colonies after Hurricanes Irma & Maria (2017) was 61.57 ± 16.86%, with a range of 46.66–83.17%. Genetic analyses performed on the main nursery in the program, was shown to contain 32 multilocus genotypes (MLGs) (Calle-Triviño et al., 2020). Genetic analyses were not performed on the secondary nurseries. *A. cervicornis* spawning within nurseries and subsequent assisted fertilization, and rearing of embryos, larva, and recruits was performed for the first time on the Island in 2015 and repeated again in 2016. A fertility rate >90% was attained, but settlers’ survival rate after 332 days was 10% (Calle-Triviño et al., 2018).

## Discussion

### Reef condition and management of coral reefs

**Figure 4:**
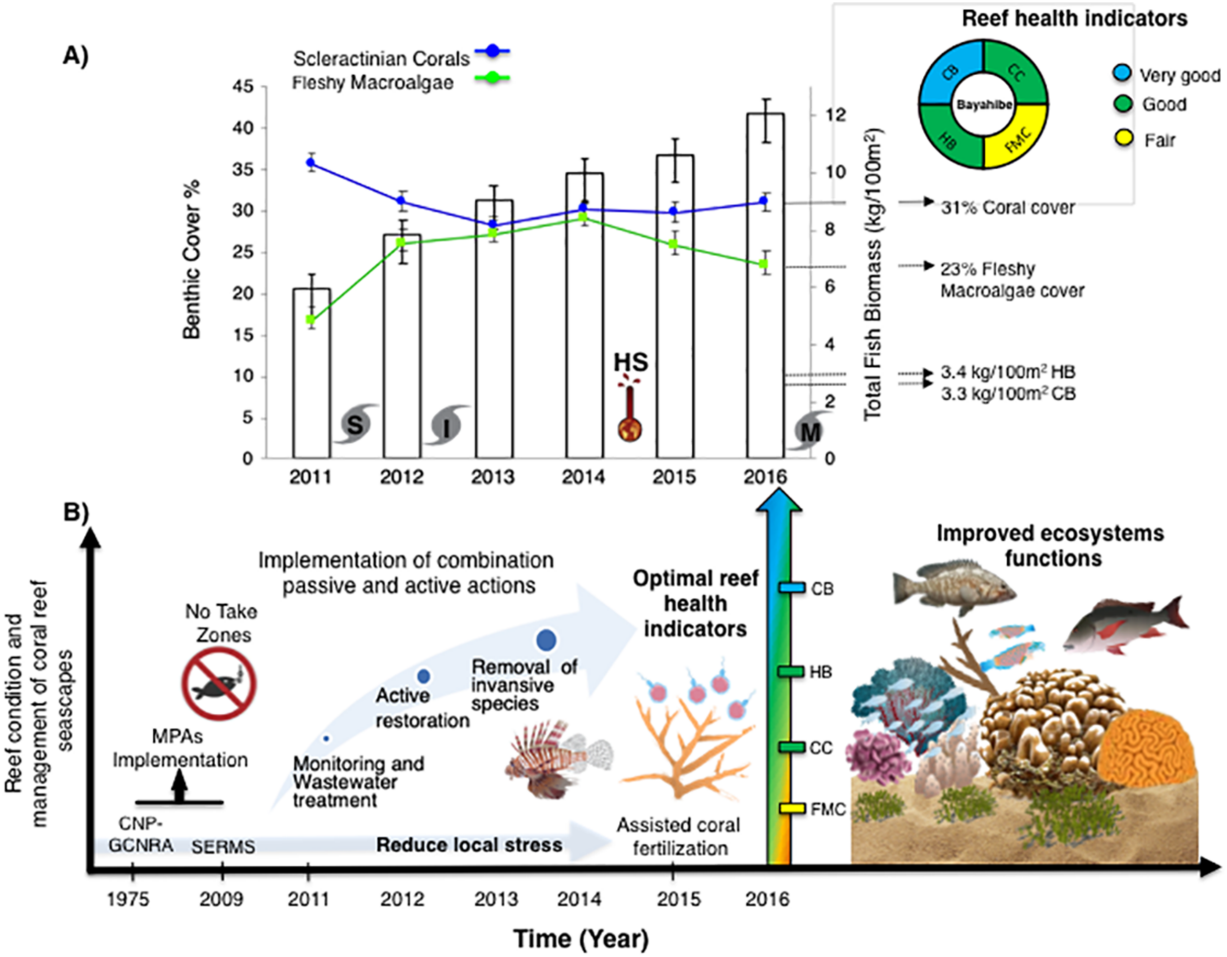
(A) Trends in the reef health indicators and reef health for 2016 in Bayahibe area. White bars represent total fish biomass (mean and standard errors). The blue line and dots show live coral cover and green line and dots represent fleshy macroalgae cover (mean and standard errors). S, Hurricane Sandy; I, Hurricane Isaac; M, Hurricane Mathew and HS, Heat stress based on distribution of the annual maximum Degree Heating Weeks (DHW; °C-weeks), (B) Optimal reef health indicators through local management of Bayahibe coral reef seascapes. CC, Coral cover; FMC, fleshy macroalgae cover; HB, herbivorous fishes biomass and CB, commercial fishes biomass. Based on the Reef Health Index (RHI) (McField et al., 2018).

**Figure 5:**
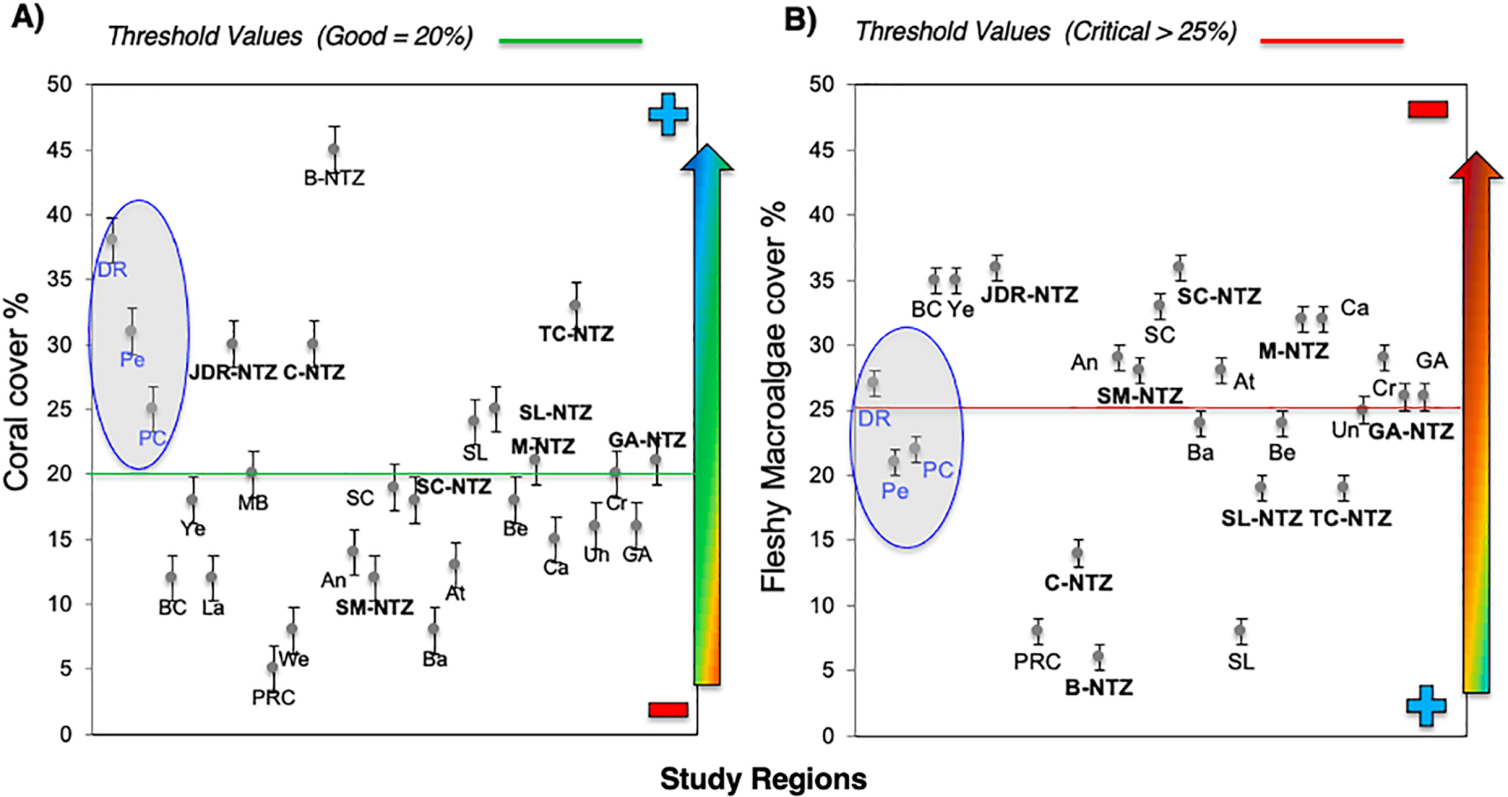
Comparison of mean (±SE), (A) coral and (B) fleshy macroalgae cover for Bayahibe Reefs (DR, Pe and PC) in blue color and the sites in Caribbean region used for comparison (Table 1). The green line corresponds to the good coral cover threshold value (>20%) and the red line critical fleshy macroalgae cover threshold value (>25%) based on the Reef Health Index (RHI) (McField et al., 2018).

Bayahibe’s reef health indicators based on the benthic cover and fish biomass show a healthy condition for 2016 (Fig. 4). The mean live coral cover of 31 ± 1.04% observed in Bayahibe, was better than other Caribbean averages (< 20% of cover; Fig. 5A). We recognize that coral cover has decreased from 2011 to 2016, however temporal analyses suggest that this decrease did not achieve significant difference. We found in our study reefs (DR 38%, Pe 31%, and PC 25%), high values of coral cover which are comparable with reefs within managed MPAs such as Bonaire (B-NTZ 45%), Cuba (JDR-NTZ 30%), Cozumel (C-NTZ 30%) or Tobago Cays (TC-NTZ 33%) (Jackson et al., 2014; Steneck et al., 2018; 2019; Martínez-Rendis, Acosta-González & Arias-González, 2020). In fact, benthos studies carried out by Steneck & Torres (2019) across the Dominican Republic, reported the most abundant coral cover to be found at two southern sites that included “La Caleta” outside of Santo Domingo, and Bayahibe; two areas that have had a long history of some form of management designation and relatively reduced human impacts. Precisely, Cortés-Useche et al. (2019) highlights that the MPA network in the SE Dominican Republic protects more than 50% of coral diversity and fosters protection of coral evolutionary history. In addition, fleshy macroalgae cover (23 ± 1.7%) was lower than other Caribbean reef averages (30%) (Fig. 5B). Despite the noticeable FMC increase from 2011 to 2016, the values were not poor or critical in comparison with threshold values (>25%) for the region (McField et al., 2018). The temporal patterns in Bayahibe’s reefs suggest that changes in the benthic community structure remain stable in contrast with other degraded reef sites with higher algal cover than coral cover (phase shift) (Jackson et al., 2014; Martínez-Rendis, Acosta-González & Arias-González, 2020). This coincides with the fact that no significant differences were observed among the years (PERMANOVA) and may provide evidence of ecosystem improvement of the Bayahibe area (Fig. 5). For example, in 2016 coral and fleshy macroalgae cover were better than baseline recorded at comparable sites in 1996 by Chiappone (2001) in southeastern Dominican reefs that showed mean fleshy macroalgae cover ranged from 52% to 80% and coral cover from 11% to 20% (Cortés-Useche et al., 2019). This idea of ecosystem stability in Bayahibe is further supported by Steneck & Torres (2019) when they also reported that live coral and macroalgae cover at El “Peñón reef” and “Tortuga” (other sites in Bayahibe with similar characteristics as our sites) had remained about the same as recorded back in 2015. These healthy trajectories may be evidence of a long-term management process that has maintained mean live coral cover stable despite observing significant declines at other similar sites.

**Figure 6:**
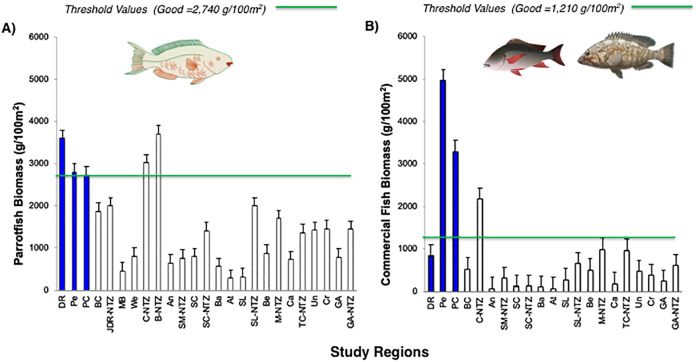
Comparison of (A) Parrotfish (Scaridae) and (B) commercial fishes (Lutjanidae and Serranidae) biomass (g/100 m^2^) for Bayahibe Reefs (DR, Pe and PC) in blue bars and sites in Caribbean region used for comparison (Table 1). The green lines correspond to the good threshold values based on the RHI (>2,740 g/100 m^2^) for parrotfish biomass and (>1,210/100 m^2^) for commercial fishes (mean and standard errors).

The noticeable increases and statistically significant temporal differences among 2011 to 2016 for total fish biomass (TB), including herbivorous fishes (Acanthuridae and Scaridae = 3.4 kg/100 m^2^) and commercial fishes (Lutjanidae and Serranidae = 3.3 kg/100 m^2^) suggest that fish assemblages in Bayahibe are in a management process under construction. Some studies indicate that certain fish species can respond to conservation actions after 10 years of protection (Molloy, McLean & Cote, 2009). According to the findings of the present study, the Bayahibe reefs can be compared with other managed fishing areas such as B-NTZ and C-NTZ that present very good threshold values (>3 kg/100 m^2^) for herbivorous fish biomass in the Caribbean region (Steneck et al., 2019; Martínez-Rendis, Acosta-González & Arias-González, 2020). Indeed, fishing regulations in these sites have been enforced since more than 10 years ago compared with unprotected sites and partially protected areas (Fig. 6). Further, management efforts in these areas also include the user fees for management of their MPAs, restrictions on urban development and visitor’s capacity limits (Perera-Valderrama et al., 2016; Steneck et al., 2019; Martínez-Rendis, Acosta-González & Arias-González, 2020). The enforcement of such actions in Bayahibe may further contribute to a reduction in anthropogenic pressure on fish populations and the seascape in general. Currently, funds and investment are limited and the funds that are available, are expected to support capacity building and enforcement of a very large area (868,900 ha). In fact, the International Union for the Conservation of Nature (IUCN, 2012) conducted a gap analysis of the legal and policy framework for protected areas in the Dominican Republic and suggested building management capacity and improving participation in all MPAs.

Most of the No-take zones and MPAs analyzed showed good reef health indicators (Jackson et al., 2014; Perera-Valderrama et al., 2016; Steneck et al., 2018, 2019; Martínez-Rendis, Acosta-González & Arias-González, 2020), including the comparison between partially protected areas in Bayahibe (DR, Pe, and PC) *vs* fished areas (Boca Chica) in the Dominican Republic (Cortés-Useche et al., 2019) (Figs. 5 and 6).

In the wider Caribbean, the decline of coral reefs condition is alarming, currently few sites can tolerate the rapid increases in sea surface temperatures (SSTs), the frequency and intensity of coral bleaching events, and local stress factors challenges (Sully et al., 2019). Region-wide, this decline has been attributed to hurricane impacts, disease outbreaks, bleaching events, or fish herbivory loss (Jackson et al., 2014). These factors put into context the importance of identifying resilient sites using long-term data. Bayahibe’s reefs have experienced lower impacts of heat stress than other regions of the Southern Caribbean, Eastern Caribbean, Southwestern Caribbean, Southern Gulf of Mexico and Western Caribbean (Muñiz-Castillo et al., 2019), or the recent impacts of the rapid spread of the stony coral tissue loss disease (SCTLD) outbreak as compared to areas such as Florida’s reefs or Mexican Caribbean (Precht et al., 2016; Alvarez-Filip et al., 2019). In addition, Bayahibe’s reefs are located on the Southeastern end of Hispaniola Island on the leeward side, with low-intensity winds and waves (Chiappone, 2001). This is an important property to maintain a healthy ecosystem, taking into account that each Caribbean site possesses differences in the disturbance regime and local history (Miyazawa et al., 2020). However, there has been a decline in reef condition caused by continuous pressures such as overfishing of herbivorous, land-based source pollution, and habitat destruction (Cramer et al., 2020). Thus, the implementation and enforcement of a combination of passive and active actions in Bayahibe, are an ideal scenario for protecting and recovering ecological dynamics and ecosystem services (Christie et al., 2009) (Fig. 4). In contrast, there are unprotected areas where several activities such as fishing, agriculture and private sector industries are allowed and unregulated (Mellin et al., 2016). For example, Miches and Boca Chica in Dominican Republic (Eastwood, Clary & Melnick, 2017; Cortés-Useche et al., 2019). MPAs from other Caribbean sites have shown low macroalgae cover as a result of the reestablishment of herbivore populations, and promoting coral cover (Mumby & Harborne, 2010; Hughes et al., 2017b). Bayahibe’s collective efforts have been made with the implementation of passive and active management, this may reflect positive feedbacks of reducing the fishing pressure with the permanent closure of fishing activities and boat transit in the “Catuano” channel since 2009. This has allowed observable stability patterns in the ecosystem structure and function, that is, no phase-shift, and increased fish biomass (herbivorous fishes), indicating that grazing pressure can be relatively constant (Arias-González et al., 2017). The fish biomass indicators (TB, HB, and CB) among 2011 to 2016 could be reflecting these positive feedbacks over time. Besides, CB showed higher values in Bayahibe in contrast with Boca Chica (unprotected), this could be related to the establishment of closed seasons by the Ministry of Environment and Natural Resources (Cortés-Useche et al., 2019). After our study, multiple regulations affecting fisheries were passed through government resolutions (Resolution No. 023−17 and No. 023−20). These regulations established either permanent or 2-year (parrot fish only) bands on the capture, possession, and commercialization of all species of sharks, rays, parrot/doctor fish and urchins living in territorial waters, as well as the trade, exports and imports of all derived products. Thus, ecological and social feedbacks may have led to positive feedback “of the grazing pressures” that underpins a healthy reef ecosystem.

### Reduction of local stressors

Coral reef systems are linked by the complex ecological relationships of coastal seascape. As such, reducing negative feedbacks becomes a necessity. Our study was primarily undertaken to analyze and correlate WQ components with reef health indicators, in areas with high tourism development. When results from WQ components were analyzed against spatio-temporal variation, no significant differences were observed for the Bayahibe area. However, the temporal dynamic by WQ factors showed pulse events. Microbiological indicators showed higher concentrations during winter months (December and January) and coincided with observed pulse events (2014) for environmental variables such as SST and NUT in this season. These pulse events also coincided with the high season for tourism in the Dominican Republic. Moreover, pulse events were also recorded in the wet season (2012 and 2015) for NUT. The higher turbidity values (NUT) in the B4 site could be due to the proximity of this site to the Dominicus reef tourism area, as a result of the high exposure to human activities that can increase sediment accumulation. In fact, these findings suggest the effects of sediment in that laden runoff can influence pulse events during rainy days (Otaño-Cruz et al., 2017). These trends of WQ variation (pulse events) have often been associated with climate variability, characterized by effects of strong precipitation in many locations in Puerto Rico, including tropical storms during the winter season (Bonkosky et al., 2009; Díaz-Ortega & Hernández-Delgado, 2014; Otaño-Cruz et al., 2017). These results may confer the high influx of coastal activities could have on adjacent reefs systems.

Water quality measurements for the study area were below legal limits (Decree 1594–84), including fecal contamination based on (APHA) and EPA recreational water quality criterion (United States Environmental Protection Agency (USEPA), 2012). In other Caribbean MPAs, for example in “Tres Palmas” Marine Reserve (TPMR) in Puerto Rico, microbiological indicators did not exceed FC counts (<130 cfu/100 mL) and <35 cfu/100 mL for ENT (Norat-Ramírez et al., 2019). In our study, lower concentrations of microbiological indicators and environmental variables were found (Table S5), these results are consistent with other managed areas, that suggest that the establishment of WQ treatment systems are helping to reduce negative feedback of land-derived stressors (Otaño-Cruz et al., 2019). Furthermore, in this study the WQ components were weakly associated with reef health changes. However, there was a group of variables (TC, ENT and NUT) that need to be observed and addressed over the long-term before they might become a problem. The rationale here is that in other Caribbean reefs, land-based sediments and pollutants have commonly been attributed to coral cover decline (Cramer et al., 2020). These datasets being accumulated, may, in the future, be used to explain appropriate effects of land-based runoff and facilitate the ability to track these changes. Long-term water quality surveys have shown explanatory variables for reef condition and therefore, would be important to continue broadening the spatio-temporal water quality sampling, and incorporate additional significantly important nutrients (Nitrogen and Phosphorus) or dissolved organic carbon (DOC) (Arias-González et al., 2016). Taking into account the importance of water quality in regulating the cycling of nutrients (Woodhead et al., 2019), maintaining and/or recovering water quality might be a potential key driver for coral recovery for Caribbean reefs (Pawlik, Burkepile & Thurber, 2016).

Multiple studies described restricting terrestrial runoff including wastewater treatment plants, as a solution to enhance coral reefs health in areas of high development (Andersson et al., 2019). Water treatment in Bayahibe’s MPA has been funded and implemented by private sector initiatives spearheaded by the tourism sector. The main goal is enhancing coastal water quality in an area where tourism activities such as snorkeling, and SCUBA predominate. In contrast there are other areas where the private sector has been hands-off and has allowed treatment plants to degrade and reefs to receive fecal pollution directly or from faulty septic tanks (Norat-Ramírez et al., 2019). For example, in Boca Chica (unprotected area) outside of Santo Domingo, wastewater plants have collapsed due to higher urban activities and lack of upkeep of the facilities (Cortés-Useche et al., 2019). In Miches located on the south side of the Samana Bay, there is no sewage or water treatment system (Clary, 2008). The lack of a treatment system has been linked to high abundances of nutrient indicator species and poor reef conditions (Eastwood, Clary & Melnick, 2017). The terrestrial and marine ecosystems linkage supporting the role of land-based protection in order to achieve good reef condition.

Knowing that the invasion of lionfish (*Pterois* spp.) poses one of the greater threats to coral reefs in the region (Morris, 2012), efforts have been taken to control lionfish populations through capture and removal of individuals. This method has been successful in reducing their densities and biomass in Bayahibe and other Caribbean sites and is considered an accepted tool to reduce localized invasions (Frazer et al., 2012; De León et al., 2013). Further, at Bayahibe, for instance, removal efforts have actively engaged the community and have been socially successful contributing to persistent catches (2,534 lionfish) from 2011 to 2016 of individuals in specific sites, even in events with low participation (2014) but with a high CPUE (Cortés-Useche et al., 2017). This result may be considered successful in terms of catch (i.e., total number of lionfish captured) and coincides with the fact that in several Caribbean Island States, derbies with higher catch are dominated by recreational divers or with a mixed public, using SCUBA diving demonstrating the success factor of community engagement and contrast with artisanal fishers (i.e., usually free-diving) (Malpica-Cruz, Chaves & Côté, 2016). This is just one example of how the community driven, capture and removal method can control invasive species based on long-term stable programs and this show the possibility in a small localized area, where annual use can be intensive during multiple consecutive years, such as sites where tournaments are periodically organized, to observe ecological changes in the abundance and biomass of the invasive species (Barbour et al., 2011). Besides, these events provide food (fishes) supporting vital nutrition to Bayahibe coastal community.

Active coral restoration through the implementation of coral nurseries and the coral gardening methodology has given artisanal fishers, tourism-related industries, non-governmental organizations (NGOs), government, and the scientific community a unique opportunities to collaborate in local restoration projects (Lirman & Schopmeyer, 2016) and providing a platform for experimental research, cognitive and experiential benefits in in the community of Bayahibe (Calle-Triviño et al., 2018; Woodhead et al., 2019; Cortés-Useche et al., 2019). Over the years, restoration training in Bayahibe has been provided to the local community including fishers, boat captains, tourism service providers, park rangers, diving instructors, divers, university students, etc. In addition, this program included interventions enabled by strong scientific and private partnerships (Bayraktarov et al., 2020). The *A. cervicornis* restoration program in the area is focused on maximizing growth rates and minimizing mortality (Edwards, 2010). Calle-Triviño et al. (2020) highlights that in Bayahibe there are enough genotypes to expand program and still maintain genetic diversity, indicating that coral nurseries could serve as genotype reservoirs better adapted to the strong environmental changes, and even as havens in the face of disease outbreaks, storms, and extreme temperatures (Schopmeyer et al., 2012; Rinkevich, 2015). The results of the program in Bayahibe under conditions of stress caused by the strong cyclonic seasons in 2016 and 2017 in the Caribbean demonstrate recovery, especially when compared to other regions of the Caribbean such as Puerto Rico (Toledo-Hernández et al., 2018). In the same way, nurseries also serve as aggregation sites for coral larvae, fishes, and other organisms (Amar & Rinkevich, 2007; Shafir & Rinkevich, 2010), contributing to overall ecosystem diversity. In this sense, coral reef restoration may play an active role for the future of the reef.

The dominant corals on Bayahibe’s reefs were the *Orbicella* spp. complex and *Agaricia agaricites*. However, Cortés-Useche et al. (2019) and Calle-Triviño et al. (2020) recorded the presence of *A. cervicornis* in nearby sites. Thus, broadening the monitoring efforts may increase information regarding the distribution and occurrence of the threatened staghorn coral *A. cervicornis*. This finding emphasizes coral composition in Bayahibe dominated by reef-building corals in contrast with other locations across the Caribbean region; dominated by species of genus *Porites* spp. which reduces the complexity of the habitat and modifying ecosystem functions (Perry et al., 2015). Accordingly, coral reefs with structural complexity may be considered as service providers of coastal protection from waves and extreme weather events.

### Bayahibe as a case study of coral reefs management

One of the main contributions of this work is the use of reef health indicators over time, taking into account multi-ecological drivers effects and the stability of alternative states in Caribbean reefs. Our reef health indicators for Bayahibe suggest that reefs within the Bayahibe reef track are in good condition, taking into account Reef Health Index (McField et al., 2018) (Fig. 4). Reef health and resilience includes several components. Thus, the implementation of passive and active actions is not the absolute solution for coral reef recovery. In this study, we observed that local management has the potential to contribute to a reduction of local stress on these reefs, which have remained stable even with a local disturbance regime characterized by low impacts of hurricanes, heat stress and outbreaks of coral diseases. The combination of these factors eventually can improve ecosystem function such as fish assemblages and coral species composition, thus, provides benefits gained from having a healthy reef ecosystem and key habitat such as important refugia for species (Woodhead et al., 2019). Also, improved local management may contribute to ecosystem services for local economies, and livelihoods (Hernández-Delgado, 2015). This includes, for the Bayahibe area, provisioning services such as food products, coastal protection from damages inflicted by natural events, and substantial economic benefits such as jobs creation, income generation and tourism activities. Hence, this puts into context that any appropriately managed network of MPAs, with interventions enabled by strong scientific and private partnerships, can improve the efficacy of interventions and provide ecosystem and socio-economic benefits for areas of high tourism development such as the southeastern Dominican Republic (Calle-Triviño et al., 2020; Bayraktarov et al., 2020). For example, in the study area, the responsible tourism movement “Wave of Change” of the Iberostar group created its land-based facility to contribute as a platform for research and environmental education (Schmidt-Roach et al., 2020).

The establishment of MPAs in the Dominican Republic is an important piece of the management process and should be a significant national policy. Recently, the government joined several global conservation initiatives and sustainable finance mechanisms. However, how MPA governance influences conservation outcomes remains undervalued. Staffing and budget capacity has been very limited in many MPAs such as the SCRMS and GCNRA, where there is reduced control and enforcement of fishing regulations (Cortés-Useche et al., 2019). However, there is one example that stands out. It is to our knowledge, that there is a signed agreement for the co-management of the SCRMS and a management and action plan is being prepared which includes the establishment of map zoning the different uses of coastal (fishing, tourism, research and conservation). Currently, the combined impact of local environmental degradation, poor governance capacity of some Caribbean island states in terms of staff capacity and financial budget is a challenge for reef managers (Gombos et al., 2011; McConney & Pena, 2012). Further, these managers have a limited toolbox on which to rely on to mitigate threats (Aswani et al., 2015). Data presented here of local management processes, and enforcement of fishing regulations for all MPAs in the study areas. However, effectiveness will depend on political and social long-term commitment, multidisciplinary approaches based on solid science using technological tools, and well-devised action plans (Bearzi, 2007; Foo & Asner, 2019). It requires compliance with several seascapes conservation-oriented key management factors (Edgar et al., 2014), such as the establishment of; (1) fishery recovery zones, (2) fishing and other recreation zones (zonification), (3) marine and coastal biological corridors between MPAs, (4) adequate MPA spatial size, and design, and (5) planning land use and management of adjacent water basins. We agree with several studies suggesting that a combination of reef rehabilitation techniques and appropriate management actions can improve their ecosystem’s recovery capacity and provide alternatives for sustainable conservation (Rinkevich, 2014; Hernández-Delgado, Mercado-Molina & Suleimán-Ramos, 2018). Restoration efforts in the area should be focused on scaling up reef-building species, rehabilitation, and the genetic study of corals resistant to heat stress and diseases (Morikawa & Palumbi, 2019).

## Conclusions

Overall, Bayahibe’s reefs show good condition based on several reef health indicators that were measured and analyzed: live coral cover, low fleshy macroalgae cover, and recovery of fish biomass (both commercial and herbivorous fishes). Our results show an example of resistant reefs in a region under rapidly increasing changes and rapid spread of coral disease outbreaks. Nevertheless, identifying and protecting persistent reefs should continue based on long-term monitoring, and broadening reef surveys. Understanding the concept of seascape connectivity and considering nature as a solution may result in a more sustainable tourism. To sustain the economy in the Dominican Republic tourism is very important, specifically for the Bayahibe area, and is a source of development for community livelihoods. Minimizing the pressure of key drivers in a region under increasing tourism development is a major challenge and seems to be working in the area. Achieving this could be improved by a strong local commitment, including the enforcement of actions that will increase ecological, economic, and social gains and serve as an example for other Caribbean zones. Therefore, continuing with the current fishing policy, improving performance and enforcement of MPAs will be key to learning lessons and demonstrating real benefits from that management process.

## Supplemental Information

10.7717/peerj.10925/supp-1 Supplemental Information 1 List of codes and characteristics of the 3 study MPAs. CNP = Cotubanama Natural Park, GCNRA = Guaraguao Catuano Natural Recreation Area and SCRMS = Southeastern Coral Reef Marine Sanctuary.

10.7717/peerj.10925/supp-2 Supplemental Information 2 Benthic cover along the spatio-temporal pattern in Bayahibe reefs. Includes data for coral and fleshy macro algae cover. DR = Dominicus Reef (Southeastern Coral Reef Marine Sanctuary), Pe = “Peñón” reef (Guaraguao Catuano Recreation Natural Area) and PC = “Punta Cacón” reef (Cotubanama Natural Park).

10.7717/peerj.10925/supp-3 Supplemental Information 3 Summary of the permutational analysis of variance (PERMANOVA) test results for benthic community. CC = Coral Cover and FMC = Fleshy Macroalgae Cover. For fish community; TB = Total fiah Biomass, HB = Herbivorous fish (Acanthuridae and Scaridae) Biomass and CFM = Commercial fishes (Lutjanidae and Serranidae) Biomas. For WQ = Water quality components; TC = Total coliforms, FC = Fecal coliforms, ENT = Enterococci, NUT = Turbidity in nephelometric turbidity units, pH and SST = sea surface temperature. Pairwise comparison between sites. DR = Dominicus Reef, Pe = “Peñón” reef, and PC = “Punta Cacón” reef. Bold p-values represent significance.

10.7717/peerj.10925/supp-4 Supplemental Information 4 Cover by Species.

10.7717/peerj.10925/supp-5 Supplemental Information 5 WQ Data.

10.7717/peerj.10925/supp-6 Supplemental Information 6 Biomass data.

10.7717/peerj.10925/supp-7 Supplemental Information 7 PCO plot of distance among centroids, to display the variations in coral species composition based on Bray-Curtis similarity matrices performed on square root-transformed data by site clusters. This model explained 55.2% of the observed spatio-temporal variation in coral species composition by site. Blue color represent DR = Dominicus Reef, red color Pe = “Peñón” reef, and green color PC = “Punta Cacón” reef. PST = *Pseudodiploria strigosa*, MCAV = *Montastraea cavernosa*, and OFAV = *Orbicella faveolata*.

10.7717/peerj.10925/supp-8 Supplemental Information 8 Control of invasive species. Bars display No. Individuals of lionfish (Pterois spp.) per year. Circles display the median of size class in cm (SE) and minimum and maximum values (black dots).

## Additional Information and Declarations

### Data Availability

The following information was supplied regarding data availability: Raw data are available in the Supplemental Files.
