# Supplementary material for: Conservation actions and ecological context: optimizing coral reef local management in the Dominican Republic
Source: PeerJ. 2021 Mar 9;9:e10925. doi: 10.7717/peerj.10925 (PMC7953877; doi:10.7717/peerj.10925)
Supplement: Supplemental Information 1 — CNP = Cotubanama Natural Park, GCNRA = Guaraguao Catuano Natural Recreation Area and SCRMS = Southeastern Coral Reef Marine Sanctuary. [file peerj-09-10925-s001.doc]

**Supplementary information “Conservation actions and ecological context: optimizing coral reef local management in the Dominican Republic”**

**Table S1**

List of codes and characteristics of the 3 study MPAs. CNP = Cotubanama Natural Park, GCNRA = Guaraguao Catuano Natural Recreation Area and SCRMS = Southeastern Coral Reef Marine Sanctuary.

| **MPA** | **IUCN** | **Site** | **Code** | **Year of legal establishment** | **Fishing** | **Dive Intensity** |
| --- | --- | --- | --- | --- | --- | --- |
| CNP | II | Punta Cacón | PC | 1975 | Regulated | 9/day |
|  |  |  |  |  |
|  |  | Peñón | Pe | 1975 | Regulated | 34/day |
| GCNRA | VI |  |  |  |  |  |
|  |  |  |  |  |  |  |
| SCRMS | I | Dominicus Reef | DR | 2009 | Regulated | 61/day |
|  |  |  |  |  |
|  |  |  |  |  |  |  |

**References**

Cortés-Useche C, Muñiz-Castillo AI, Calle-Triviño J, Yathiraj R, Arias-González JE. 2019. Reef condition and protection of coral diversity and evolutionary history in the marine protected areas of Southeastern Dominican Republic. Regional Studies in Marine Science 32:100893. DOI: 10.1016/j.rsma.2019.100893.
