# Supplementary material for: Conservation actions and ecological context: optimizing coral reef local management in the Dominican Republic
Source: PeerJ. 2021 Mar 9;9:e10925. doi: 10.7717/peerj.10925 (PMC7953877; doi:10.7717/peerj.10925)
Supplement: Supplemental Information 2 — DR = Dominicus Reef (Southeastern Coral Reef Marine Sanctuary), Pe = “Peñón” reef (Guaraguao Catuano Recreation Natural Area) and PC = “Punta Cacón” reef (Cotubanama Natural Park). [file peerj-09-10925-s002.doc]

**Supplementary information “Conservation actions and ecological context: optimizing coral reef local management in the Dominican Republic”**

**Table S1. Benthic cover along the spatio-temporal pattern in Bayahibe reefs. Includes data for coral and fleshy macro algae cover. DR = Dominicus Reef (Southeastern Coral Reef Marine Sanctuary), Pe = “Peñón” reef (Guaraguao Catuano Recreation Natural Area) and PC = “Punta Cacón” reef (Cotubanama Natural Park).**

| **Site** | **Coral cover %** | | | | | | | | | | | |  |
| --- | --- | --- | --- | --- | --- | --- | --- | --- | --- | --- | --- | --- | --- |
|  | **2011** | | **2012** | | **2013** | | **2014** | | **2015** | | **2016** | |  |
| DR | | 39.5 | | 35.5 | | 31.0 | | 35.5 | | 38.2 | | 37.5 | |
| Pe | | 38.4 | | 32.5 | | 31.3 | | 31.3 | | 30.0 | | 31.2 | |
| PC | | 29.3 | | 25.3 | | 22.3 | | 23.7 | | 21.2 | | 24.5 | |
|  | | **Fleshy macroalgae cover %** | | | | | | | | | | | |
|  | | **2011** | | **2012** | | **2013** | | **2014** | | **2015** | | **2016** | |
| DR | | 18.7 | | 27.1 | | 31.5 | | 24.8 | | 24.0 | | 27.0 | |
| Pe | | 16.8 | | 17.7 | | 24.0 | | 32.0 | | 26.5 | | 21.1 | |
| PC | | 14.8 | | 33.6 | | 26.3 | | 30.8 | | 27.0 | | 22.1 | |
