# Supplementary material for: Conservation actions and ecological context: optimizing coral reef local management in the Dominican Republic
Source: PeerJ. 2021 Mar 9;9:e10925. doi: 10.7717/peerj.10925 (PMC7953877; doi:10.7717/peerj.10925)
Supplement: Supplemental Information 3 — CC = Coral Cover and FMC = Fleshy Macroalgae Cover. For fish community; TB = Total fiah Biomass, HB = Herbivorous fish (Acanthuridae and Scaridae) Biomass and CFM = Commercial fishes (Lutjanidae and Serranidae) Biomas. For WQ = Water quality components; TC = Total coliforms, FC = Fecal coliforms, ENT = Enterococci, NUT = Turbidity in nephelometric turbidity units, pH and SST = sea surface temperature. Pairwise comparison between sites. DR = Dominicus Reef, Pe = “Peñón” reef, and PC = “Punta Cacón” reef. Bold p-values represent significance. [file peerj-09-10925-s003.doc]

**Supplementary information “Conservation actions and ecological context: optimizing coral reef local management in the Dominican Republic”**

**Table S2.** Summary of the permutational analysis of variance (PERMANOVA) test results for benthic community; CC = Coral Cover and FMC = Fleshy Macroalgae Cover. For fish community; TB = Total fiah Biomass, HB = Herbivorous fish (Acanthuridae and Scaridae) Biomass and CFM = Commercial fishes (Lutjanidae and Serranidae) Biomas. For WQ = Water quality components; TC = Total coliforms, FC = Fecal coliforms, ENT = Enterococci, NUT = Turbidity in nephelometric turbidity units, pH and SST = sea surface temperature. Pairwise comparison between sites. DR = Dominicus Reef, Pe = “Peñón” reef, and PC = “Punta Cacón” reef. Bold p-values represent significance.

| **Source** | **TEST** | **Factor** | **df** | **Pseudo-F** | **P (perm)** |
| --- | --- | --- | --- | --- | --- |
| **Benthic** |  |  |  |  |  |
| CC | PERMANOVA | year | 5 | 21,294 | 0.139 |
|  |  | site | 2 | 25.79 | **0.001** |
|  |  | 2011 vs 2016 | 1 | 3.076 | 0.875 |
|  | PAIR-W-TEST | DR vs Pe | 1 | 2.5646 | 0.064 |
|  |  | DR vs PC | 1 | 5.4023 | **0.006** |
|  |  | Pe vs PC | 1 | 7.3224 | **0.001** |
| FMC | PERMANOVA | year | 5 | 0.38548 | 0.869 |
|  |  | site | 2 | 2,302 | 0.144 |
|  |  | 2011 vs 2016 | 1 | 4,668 | **0.0334** |
| **Fish** |  |  |  |  |  |
| TB | PERMANOVA | year | 5 | 24,084 | 0.076 |
|  |  | site | 2 | 19,314 | 0.157 |
|  |  | 2011 vs 2016 | 1 | 34 | **0.0001** |
| HB |  | 2011 vs 2016 | 1 | 18.58 | **0.0007** |
| CB |  | 2012 vs 2016 | 1 | 6.902 | **0.0153** |
| **WQ** |  |  |  |  |  |
|  | PERMANOVA | year | 5 | 1.656 | 0.1253 |
|  |  | site | 2 | 0.6628 | 0.632 |
|  |  | Season | 1 | 1.118 | 0.3153 |
| Microbiological indicators |  | Se x TC | 1 | 1.326 | 0.1248 |
|  |  | Se x FC | 1 | 1.89 | **0.0271** |
|  |  | Se x ENT | 1 | 1.36 | 0.1499 |
| Environmental Variables |  | Se x NUT | 1 | 6.438 | **0.0001** |
|  |  | Se x pH | 1 | 13.89 | **0.0001** |
|  |  | Se x SST | 1 | 78.53 | **0.0001** |
| **Lion Fish** |  |  |  |  |  |
| Abundance | PERMANOVA | year | 5 | 0.417 | 0.834 |
