# Supplementary figures and images for: Conservation actions and ecological context: optimizing coral reef local management in the Dominican Republic
Source: PeerJ. 2021 Mar 9;9:e10925. doi: 10.7717/peerj.10925 (PMC7953877; doi:10.7717/peerj.10925)

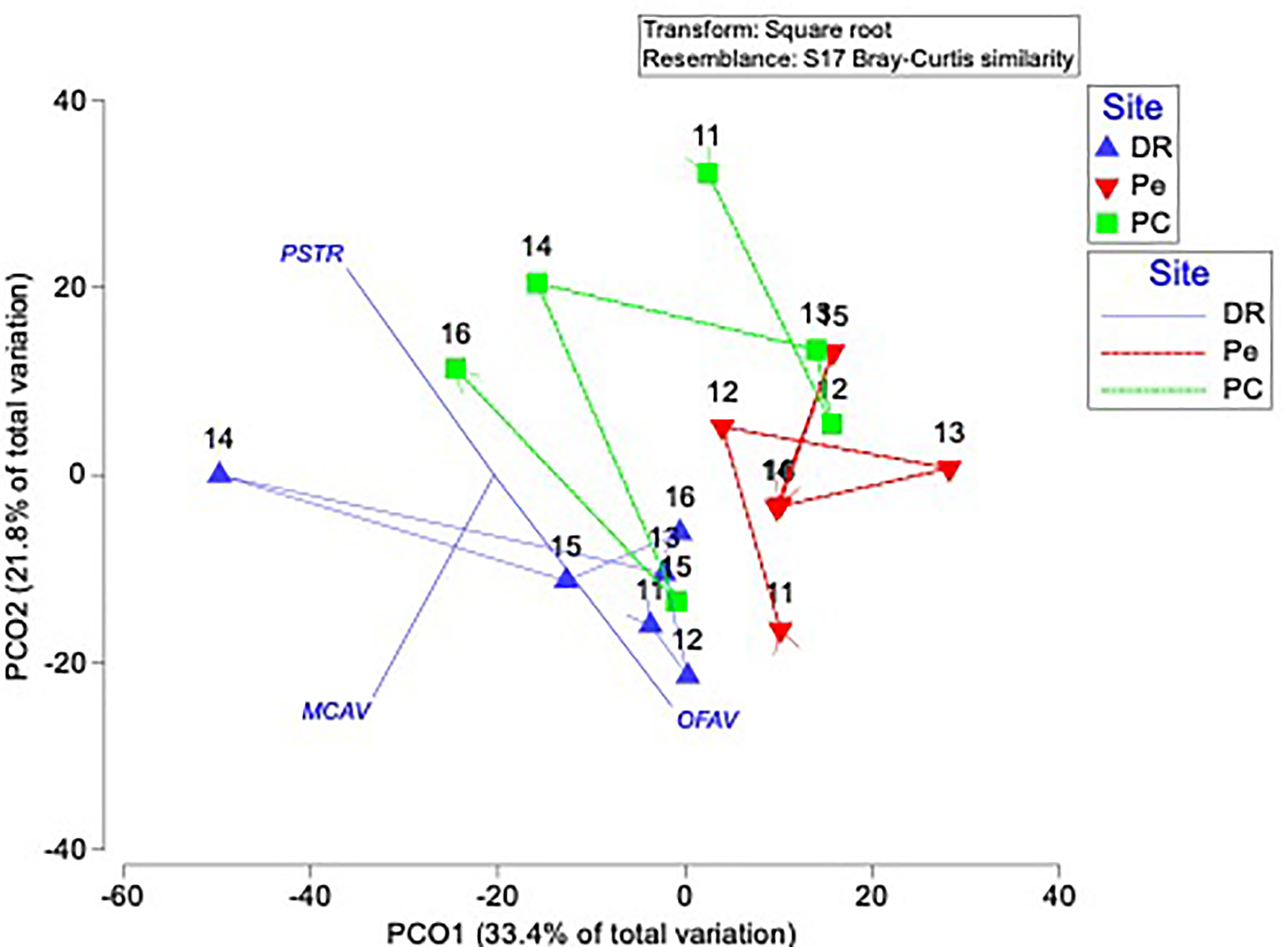

Supplement: Supplemental Information 7 — This model explained 55.2% of the observed spatio-temporal variation in coral species composition by site. Blue color represent DR = Dominicus Reef, red color Pe = “Peñón” reef, and green color PC = “Punta Cacón” reef. PST = Pseudodiploria strigosa, MCAV = Montastraea cavernosa, and OFAV = Orbicella faveolata. [file peerj-09-10925-s007.png]

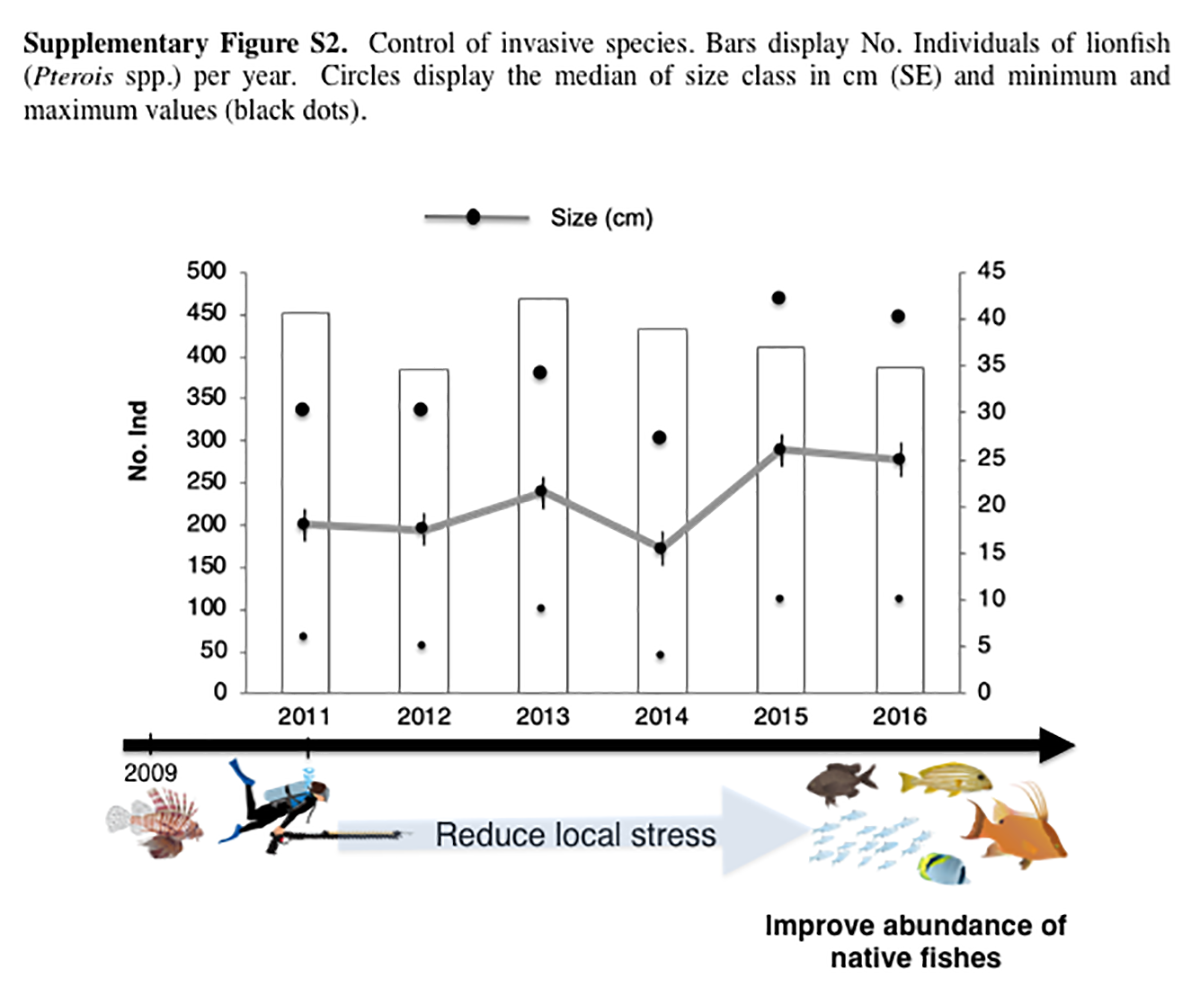

Supplement: Supplemental Information 8 — Circles display the median of size class in cm (SE) and minimum and maximum values (black dots). [file peerj-09-10925-s008.png]
